# Apical periodontitis: preliminary assessment of microbiota by 16S rRNA high throughput amplicon target sequencing

**DOI:** 10.1186/s12903-018-0520-8

**Published:** 2018-04-02

**Authors:** Federico Mussano, Ilario Ferrocino, Natalija Gavrilova, Tullio Genova, Alessandro Dell’Acqua, Luca Cocolin, Stefano Carossa

**Affiliations:** 1 CIR Dental School, Department of Surgical Sciences, University of Turin, via Nizza 230, 10126 Turin, Italy; 2 DISAFA - Microbiology and Food Technology sector, University of Turin, Largo Paolo Braccini n°2, 10095 Grugliasco, TO Italy; 3 Department of Life Sciences and Systems Biology, University of Turin, Turin, Italy; 4 Azienda Ospedaliero-Universitaria Città della Salute, San Giovanni Battista di Torino, Turin, Italy

**Keywords:** Periapical granulomas (PGs), Radicular cysts (RCs), Apical periodontitis (AP), Microbiota, High throughput amplicon target sequencing

## Abstract

**Background:**

Apical periodontitis includes periapical granulomas and radicular cysts, which are histologically distinguished by the absence and the presence of an epithelial lining, respectively. The main cause of apical periodontitis is the bacterial colonization of the root canal space. This research aimed at assessing whether and how periapical granulomas and radicular cysts differ in terms of microbiota using high throughput amplicon target sequencing (HTS) techniques.

**Methods:**

This study included 5 cases of Periapical Granulomas (PGs) and 5 cases of Radicular Cysts (RCs) selected on the base of histology out of 37 patients from January 2015 to February 2016. Complete medical history, panoramic radiograms (OPTs) and histologic records of each patient were assessed. Only lesions greater than 1 cm in diameter and developed in proximity to teeth with bad prognosis were included. The microbiota present in periapical granulomas and radicular cysts thus retrieved was finely characterized by pyrosequencing of the 16S rRNA genes.

**Results:**

The core of OTUs shared between periapical granulomas and radicular cysts was dominated by the presence of facultative anaerobes taxa such as: *Lactococcus lactis*, *Propionibacterium acnes*, *Staphylococcus warneri, Acinetobacter johnsonii* and *Gemellales*. *L. lactis,* the main OTUs of the entire datasets, was associated with periapical granuloma samples. Consistently with literature, the anaerobic taxa detected were most abundant in radicular cyst samples. Indeed, a higher abundance of presumptive predicted metabolic pathways related to Lipopolysaccharide biosynthesis was found in radicular cyst samples.

**Conclusions:**

The present pilot study confirmed the different microbial characterization of the two main apical periodontitis types and shade light on the possible role of *L. lactis* in periapical granulomas.

## Background

Apical periodontitis (AP) is associated with endodontically involved teeth [1, 2]. In most cases, it is impossible to distinguish between periapical granulomas (PGs) and radicular cysts (RCs), without recurring to biopsy [3]. The occurrence of periapical granulomas ranges between 9.3 and 87.1% [4]. Radicular cysts are believed to form by proliferation of the epithelial cell rests of Malassez in inflamed periradicular tissues [5]. Whether they be pocket cysts (cavity open to the root canal) or true cysts (completely enclosed by lining epithelium) [6], their reported incidence among periapical lesions varies from 6% to 55% [7]. As radiography for determining APs has been questioned for scientific investigations [8], differential diagnosis is possible only with histopathological examination [9]. Whatever the diagnosis, root canal debridement is the first choice treatment of APs [10, 11].

Bacterial colonization of the root canal space has been demonstrated as the main etiologic factor of APs [12, 13]. In two paradigmatic studies by Ricucci & Siqueira [13, 14], bacterial biofilm varied and no unique pattern for endodontic infections was identified. Bacteria could modify the severity and prognoses of APs and yet, surprisingly, little information is available in the scientific literature comparing the microbiota within PGs and RCs. The application of high throughput amplicon target sequencing (HTS) to study the microbial ecology has been witnessed over the past couple of years aimed at estimating the microbial diversity in different ecosystems using 16S rRNA gene as the target. The HTS provides an unprecedented greater sampling depth and allows the detection not only of the dominant community members but also of low-abundance taxa [15].

Flurry of research has been carried out in past decades to assess the microbiota of the human oral cavity, as well as the endodontic microbiome [14, 16, 17]. Hence, the purpose of this study was to finely characterize the microbiota present in histologically determined PGs and RCs by pyrosequencing their 16S rRNA genes. An in depth analysis of the PGs and RCs microbiota is required for a better understanding of the bacterial taxa involved with the inflammation process.

## Methods

### Study design and patients

The study was planned and performed in accordance with the Declaration of Helsinki and was approved by the Ethics committee of the Dental School, University of Turin. From January 2015 to February 2016, 121 patients with apical periodontitis were referred to the Triage of the Dental School of the University of Turin. Complete medical history and panoramic radiograms (OPTs) of each patient were assessed seeking large Apical periodontits (APs) that clearly had developed in proximity to teeth with bad prognosis. In the Oral Surgery Department, 37 patients were selected by applying the following exclusion criteria: systemic or local disease or condition (hematologic diseases, uncontrolled diabetes, serious coagulopathies, history of intravenous therapy with bisphosphonates, and/or diseases of the immune system) possibly precluding oral surgical intervention; immunosuppression; HIV+, HCV+, HBV+, TBC+, corticosteroid treatment, pregnancy, radiotherapy to the head or neck region within 12 months before surgery. A further restriction was achieved excluding teeth a) periodontally compromised, b) with endodontic communication to the oral cavity and c) vertical tooth fractures. Finally, after giving their informed consent, 10 patients underwent surgery. By careful manipulation, the periapical lesions were harvested sterilely and fixed in 4% formalin, while the hopeless teeth were extracted. Based on histology and a dimensional cut-off (lesion greater than 1 cm in diameter), among the apical periodontal lesions analyzed, 5 Radicular Cysts (RCs) and 5 Periapical Granulomas (PGs) were retrieved, dependently on the clear presence or absence of cavity lining epithelium, respectively.

### Histological analysis

The histological specimens retrieved were fixed in 4% formalin for 24 h and subsequently embedded with paraffin wax and cut into 3 μm thick sections, using a motorized microtome. Polylysine coated slides were used to enhance the adhesion of the tissue section during staining procedures. The histological structure of the lesions was assessed by traditional hematoxylin and eosin staining for optical microscopy.

### DNA analysis by pyrosequencing

Two slices of formalin-fixed, paraffin-embedded (FFPE) tissue samples (about 10 mg of tissue) were used for total genomic DNA extraction. Samples were pre-treated at 55 °C for a minimum of 1 h with the dissolving buffer and Proteinase K (20 mg/ml) according to the manufacturer’s instructions (Bi*O*stic® FFPE Tissue DNA Isolation Kit Mobio, Carlsbad, CA, USA). DNA was used to study the microbial diversity by pyrosequencing of the amplified V1–V3 region of the 16S rRNA gene, recurring to the primers Gray28F (5’-TTTGATCNTGGCTCAG) and Gray519r (5’-GTNTTACNGCGGCKGCTG) that amplify a fragment of 520 bp, following PCR conditions previously reported [17]. PCR products were purified twice with Agencourt AMPure purification kit (Beckman Coulter, Milan, Italy), and quantified using the PlateReader AF2200 (Eppendorf, Hamburg, Germany) with PicoGreen assay and an equimolar pool was obtained prior to further processing. Due to poor DNA quality, PG_8 sample was excluded. The amplicon pool was used for pyrosequencing on a GS Junior platform (454 Life Sciences, Roche, Monza, Italy) according to the manufacturer’s instructions by using Titanium chemistry.

### Bioinformatics analysis and metagenomic prediction

QIIME 1.9.0 software was employed to analyze 16S rRNA data [18]: OTUs (operational taxonomic units) were picked at 99% of similarity by means of UCLUST clustering methods [19]. Representative sequences from each cluster were used to assign taxonomy through matching against the Greengenes 16S rRNA gene database version 2013 by the RDP classifier. R environment (www.r-project.org) was adopted to elaborate statistics as well as plotting. To calculate the microbiota alpha diversity the authors chose the “*diversity”* function of the *vegan* package of R. Weighted UniFrac distance matrices as obtained through QIIME were imported in R to generate PCoA (Principal Coordinates Analysis) plots. Weighted UniFrac distance matrices were also used to perform ADONIS and ANOSIM statistical tests owing to compare_categories.py script of QIIME. OTU tables, which were filtered at 0.2% abundance in at least two samples, were used to compare each OTU based on the passed sample groupings (PGs and RCs) through the group_significance.py script of QIIME. OTUs co-occurrence co-exclusion was carried out by the *psych* package of R (www.r-project.org) and it was further visualized through the *corrplot* package of R [20]. In order to predict the inferred metagenome, the authors used PICRUSt [21] so as to predict abundances of gene families based on 16S rRNA sequences data [22]. Briefly, the pick OTUs step was re-performed at 97% similarity against the Greengenes database and the resulting KEGG orthologs table was then collapsed at level 3 of the KEGG annotations in order to display the inferred metabolic pathways. The resulting table was imported in the GAGE Bioconductor package [23] to identify biological pathways overrepresented or underrepresented between PGs and RCs samples. To characterize the accuracy of PICRUSt, the Nearest Sequenced Taxon Indexes (NSTI) were calculated [21]. All the sequencing data were deposited at the Sequence Read Archive of the National Center for Biotechnology Information (accession numberSRP096711).

## Results

APs were subdivided into PGs and RCs on the base of the histological analysis performed on the biopsy samples. The relevant data concerning patients’ age and gender are reported in Table 1, along with dimensions and region of the lesion. Also any previous canal root therapy was recorded. It is to be noted that all patients were older than 18 years and were in good health conditions as per inclusion criteria (Table 1).

**Table 1:** Salient data retrieved form the patient’s records

Sample_code	gender	age	Histology	Maximum diameter	Region^a^	Root canal therapy^b^
RC_1	M	28	RC	21 mm	4.6	+ 4.6
RC_2	F	30	RC	20 mm	3.6	–
RC_3	M	31	RC	24 mm	4.6	+ 4.6
RC_4	M	29	RC	22 mm	1.1, 1.2, 1.3	+ 1.1
RC_5	M	40	RC	13.5 mm	4.6	–
PG_6	F	23	PG	20 mm	1.6, 1.7 1.8	+ 1.6, 1.7, 1.8
PG_7	F	39	PG	20 mm	3.6	–
PG_8	F	25	PG	15 mm	4.7	+ 4.7
PG_9	M	36	PG	20 mm	2.6, 2.7	+ 2.6, 2.7
PG_10	F	20	PG	11 mm	3.8	–

^a^ “Region” refers to the tooth/teeth adjacent to the APs

^b^ “Root canal therapy” indicates if tooth/teeth received endodontic treatment before the extraction

### Microbial diversity

A total of 163.832 raw reads were obtained after the sequencing. 65.070 reads passed the filters applied through QIIME, with an average value of 7.230 reads/sample and a sequence length of 486 bp. The rarefaction analysis and the estimated sample coverage (Table 2) indicated that there was a satisfactory coverage for all the samples (ESC > 96%). The richness of the samples varied from a minimum of 22 to a maximum of 202 OTUs. (Table 2) Alpha-diversity indices (Table 2) showed no difference on the level of complexity (*P* > 0.05) of RCs samples compared to PGs.

**Table 2:** Number of observed diversity and estimated sample coverage (ESC) for 16S rRNA amplicons analyzed

Sample^a^	OTUs	ESC	chao1	Shannon Index
RC_1	202.00	1.00	209.46	4.48
RC _2	67.00	0.99	80.13	2.21
RC _3	164.00	1.00	171.16	2.24
RC _4	86.00	0.99	109.40	2.86
RC _5	143.00	1.00	155.83	2.34
PG_6	129.00	1.00	135.00	2.58
PG_7	49.00	0.99	56.33	1.90
PG_9	22.00	0.96	61.00	2.29
PG_10	49.00	0.97	76.60	2.34

^a^Samples are labeled according to type Periapical Granuloma (PG) and Radicular Cyst (RC)

**Fig. 1:**
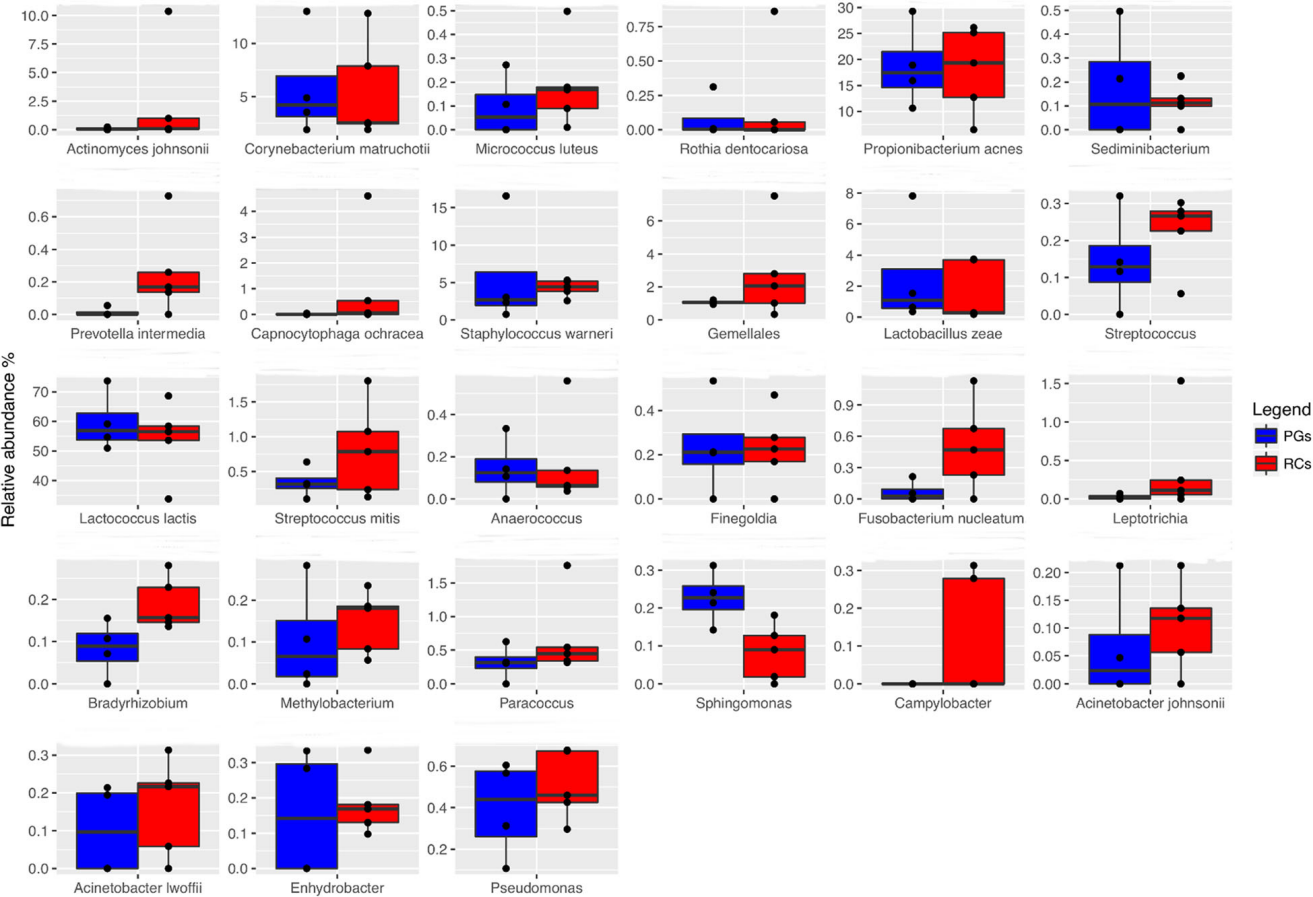
Abundance (%) of the major taxonomic groups detected by pyrosequencing. Only OTUs with an incidence above 0.2% are shown. Boxes represent the interquartile range (IQR) between the first and third quartiles, and the line inside represents the median (2nd quartile). Whiskers denote the lowest and the highest values within 1.56 IQR from the first and third quartiles, respectively. Circles represent outliers beyond the whiskers. Boxes are color coded according to the type Periapical Granulomas (PGs) blue and Radicular Cysts (RCs) red

**Fig. 2:**
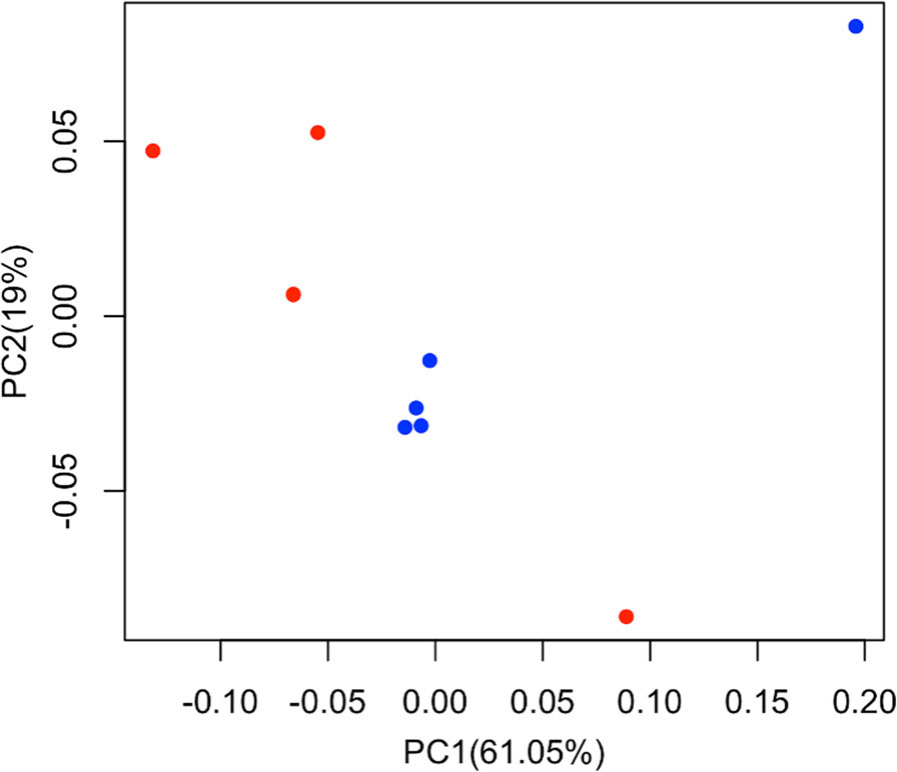
Principal Coordinate Analysis (PCoA) based on Weighted Unifrac distance matrix. Samples are color coded according of the type: Periapical Granulomas (PGs) red and Radicular Cysts (RCs) blue

In Fig. 1, the box plot shows the OTUs with a relative abundance of 0.2% in at least two samples (Fig. 1). The data showed a varied microbiota composition characterized by the presence of 40% of facultative microbes , 37% of anaerobes and 22% of aerobe microbes (Table 3). In details, the samples were characterized by the predominance of *Lactococcus lactis* (55% of the relative abundance), *Propionibacterium acnes* (18%), *Corynebacterium matruchotii* (5.5%), *Staphylococcus warneri* (5%), *Gemellales* (2%), *Actinomyces johnsonii* (2%), and *Lactobacillus zeae* (2.5%). Through principal coordinate analysis (PCoA) with a weighted UniFrac distance matrix, it was possible to show that samples from RCs grouped together and that they were well separated from PGs on the basis of their microbiota (Fig. 2); ADONIS and ANOSIM statistical tests confirmed this difference (*P* < 0.001). The differential abundance analysis showed a higher abundance (Bonferroni corrected *P* value of < 0.001) of several minor OTUs in RCs compared to PGs samples. In particular, it was possible to observe the most abundant presence of several facultative anaerobes or anaerobe OTUs such *P. acnes, Gemellales*, *Capnocytophaga ochracea*, *Paracoccus*, *Fusobacterium nucleatum*, *Prevotella intermedia* and *Rothia dentocariosa.* However *L. lactis* was found significantly more abundant (*P* < 0.001) in PGs samples than in RCs samples.

**Table 3:** Incidence of the major taxonomic groups detected by 16S rRNA amplicon target sequencing sorted by OTUs value^a^

OTUs	RCs	SD	Type	OTUs	PGs	SD	Type
*Lactococcus lactis*	54.20	12.73	Facultative anaerobe	*Lactococcus lactis*	59.63	9.97	Facultative anaerobe
*Propionibacterium acnes*	17.96	8.37	Facultative anaerobe	*Propionibacterium acnes*	18.68	7.84	Facultative anaerobe
*Corynebacterium matruchotii*	5.51	4.76	Facultative anaerobe	*Corynebacterium matruchotii*	5.83	4.96	Facultative anaerobe
*Staphylococcus warneri*	4.27	1.13	Facultative anaerobe	*Staphylococcus warneri*	5.67	7.32	Facultative anaerobe
*Gemellales*	2.75	2.83	Anaerobe	*Lactobacillus zeae*	2.59	3.52	Facultative anaerobe
*Actinomyces johnsonii*	2.31	4.52	Facultative anaerobe	*Gemellales*	1.06	0.11	Anaerobe
*Lactobacillus zeae*	1.63	1.92	Facultative anaerobe	*Pseudomonas*	0.40	0.23	Aerobic
*Capnocytophaga ochracea*	1.04	2.00	Facultative anaerobe	*Streptococcus mitis*	0.35	0.22	Facultative anaerobe
*Streptococcus mitis*	0.81	0.68	Facultative anaerobe	*Paracoccus*	0.31	0.26	Anaerobe
*Paracoccus*	0.68	0.61	Anaerobe	*Finegoldia*	0.24	0.22	Anaerobe
*Pseudomonas*	0.51	0.17	Aerobic	*Sphingomonas*	0.23	0.07	Aerobic
*Fusobacterium nucleatum*	0.50	0.43	Anaerobe	*Sediminibacterium*	0.18	0.24	Facultative anaerobe
*Leptotrichia*	0.39	0.65	Anaerobe	*Anaerococcus*	0.15	0.14	Anaerobe
*Prevotella intermedia*	0.26	0.28	Anaerobe	*Enhydrobacter*	0.15	0.18	Anaerobe
*Finegoldia*	0.23	0.17	Anaerobe	*Streptococcus*	0.14	0.13	Facultative anaerobe
*Streptococcus*	0.23	0.10	Facultative anaerobe	*Acinetobacter lwoffii*	0.10	0.12	Aerobic
*Bradyrhizobium*	0.19	0.06	Aerobic	*Methylobacterium*	0.10	0.13	Anaerobe
*Micrococcus luteus*	0.19	0.19	Aerobic	*Actinomyces johnsonii*	0.09	0.11	Facultative anaerobe
*Enhydrobacter*	0.18	0.09	Anaerobe	*Micrococcus luteus*	0.09	0.13	Aerobic
*Rothia dentocariosa*	0.18	0.38	Anaerobe	*Bradyrhizobium*	0.08	0.07	Aerobic
*Anaerococcus*	0.17	0.22	Anaerobe	*Rothia dentocariosa*	0.08	0.15	Anaerobe
*Acinetobacter lwoffii*	0.16	0.13	Aerobic	*Fusobacterium nucleatum*	0.07	0.10	Anaerobe
*Methylobacterium*	0.15	0.08	Anaerobe	*Acinetobacter johnsonii*	0.06	0.10	Aerobic
*Campylobacter*	0.12	0.16	Facultative anaerobe	*Leptotrichia*	0.03	0.03	Anaerobe
*Sediminibacterium*	0.11	0.08	Facultative anaerobe	*Capnocytophaga ochracea*	0.01	0.02	Facultative anaerobe
*Acinetobacter johnsonii*	0.10	0.08	Aerobic	*Prevotella intermedia*	0.01	0.03	Anaerobe
*Sphingomonas*	0.08	0.08	Aerobic	*Campylobacter*	0.00	0.00	Facultative anaerobe

^a^Only OTUs with an incidence above 0.2% in at least 2 samples are shown. Abundances of OTUs for each dataset (PGs and RCs) are displayed as average and standard deviations (SD)

**Fig. 3:**
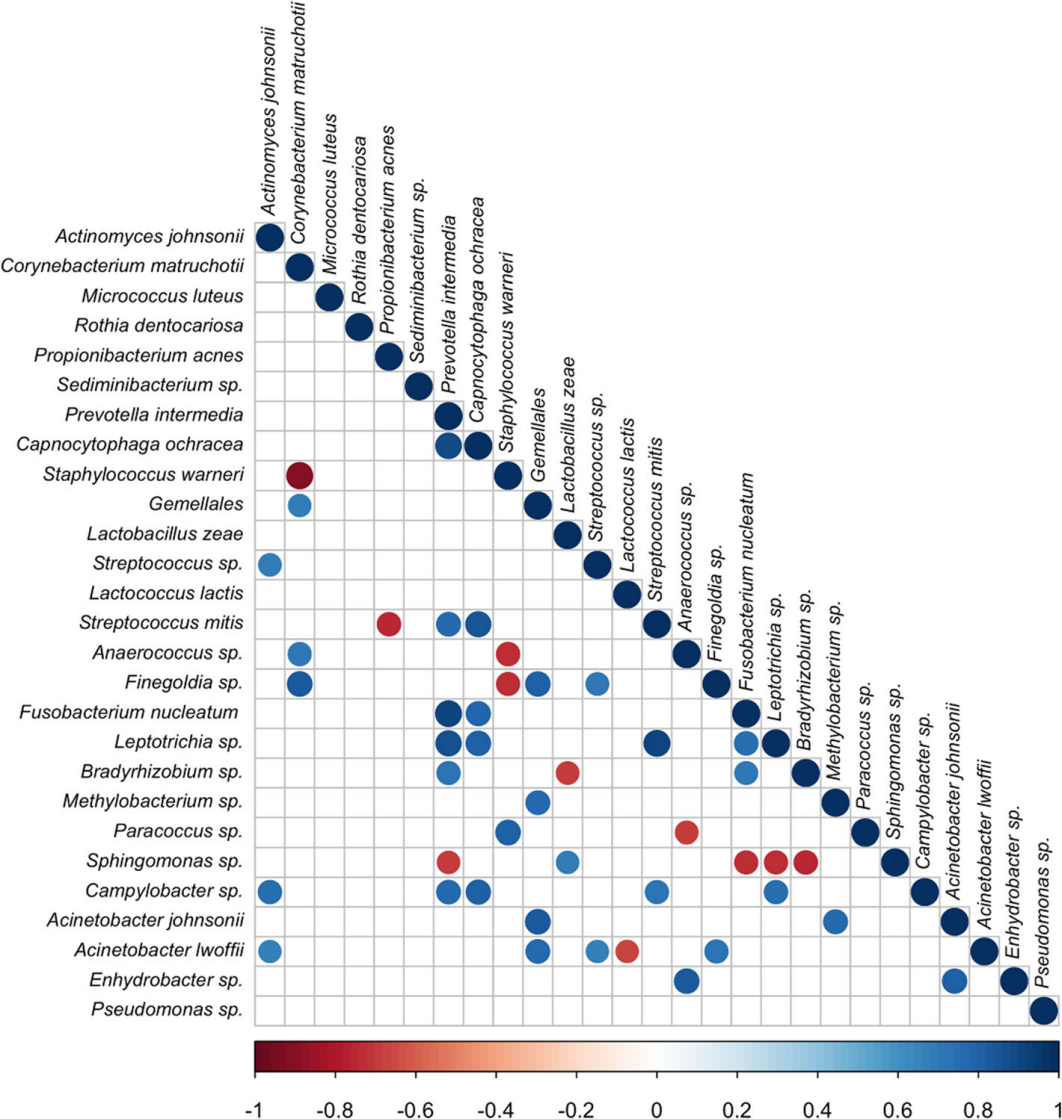
Significant co-occurrence and co-exclusion relationships between bacterial OTUs. Spearman’s rank correlation matrix of OTUs with > 0.2% abundance in at least 2 samples. The colors of the scale bar denote the nature of the correlation, with 1 indicating a perfectly positive correlation (dark blue) and − 1 indicating a perfectly negative correlation (dark red) between two microbial OTU. Only significant correlations (FDR < 0.05) are shown

The OTU co-occurrence was investigated by considering the species-level taxonomic assignment and significant correlations at false-discovery rate [FDR], < 0.05. (Fig. 3). *P. intermedia* showed the highest number of positive correlations including those with *Streptococcus mitis* and *F. nucleatum* and a co-exclusion with *Sphyngomonas* sp. Moreover the most significant OTUs in cyst samples such as *Gemellales, C. ochracea,* showed the highest number of positive correlation. The core OTUs *L. lactis* co-exclude the presence of *Acinetobacter lwoffii*, while *P. acnes* co-exclude the presence of *S. mitis*. (Fig. 3).

Regarding the predicted metagenomes, the weighted nearest-sequenced-taxon index (NSTI) for the samples, expressed as the mean SD, was 0.042 ± 0.004. Thus, a NSTI score of 0.042 indicates a satisfactory accuracy for all of the samples (96%). The pathway enrichment analysis (performed by GAGE) of the predicted metagenomes showed an enrichment of metabolic pathways such as Biosynthesis of amino acids (ko01230), Pyruvate metabolism (ko00620), Propanoate metabolism (ko00640) in PGs samples compared to RCs samples (data not shown). In contrast, from RCs samples only pathways involved in cellular processes, biosynthesis of secondary metabolites, and genes involved in Lipopolysaccharide biosynthesis (ko00540) were found.

## Discussion

The oral cavity is exposed to the external environment and is, therefore, one of the most important ways of microbial entry into the human body [17]. By invading the adjacent tissues, bacteria may induce an immune response resulting in inflammatory manifestations such as apical periodontitis [24, 25]. The presence of bacteria in PGs and RCs was previously confirmed [26]. Recently, by culture dependent methods [27], RCs were clearly demonstrated to possess a great variety of anaerobic and facultative anaerobic microbial taxa. Our results showed that the core of OTUs shared between PGs and RCs was dominated by the presence of facultative anaerobes taxa such as: *L. lactis*, *P. acnes*, *S. warneri, A. johnsonii* and *Gemellales*. In particular, *P. acnes,* reported as the most commonly detected bacterium, has been studied due to its capacity to induce the differentiation of T lymphocytes into CD25 regulatory bright cells with a potentially inhibitory effect on the immune response [24]. Actinomyces species have been implicated frequently as a cause of endodontic failure because of their ability to persist in periapical tissues [27–30].

These species are all normal commensals of the human oral cavity and were isolated in radicular cyst [25]. Beta diversity calculation as well as ADONIS and ANOSIM statistical tests display a degree of separation of the samples due to the relative abundance of the minor OTUs. Of course, within the oral cavity, many initial interactions between food microbes and human microbiota occur. Our results revealed the presence of taxa clearly derived from food like *L. lactis,* a non pathogenic taxon, usually not associated with the oral microbiota. Although the genus *Lactococcus* had already been isolated by culture dependent methods from PGs [24], here we show, for the first time, that *L. lactis* was the main OTUs of the entire datasets and it was associated with PGs samples.

It may be noteworthy that the anaerobic taxa detected were most abundant in RCs samples. *F. nucleatum*, *P. intermedia* and *R. dentocariosa* were observed to be statistically more abundant in those samples and, as previously reported, the presence of these taxa suggested the onset of secondary infection [27]. In addition, Iatrou et al. [31] determined in their study that the isolated bacteria were mostly anaerobic. Aerobe and facultative anaerobic bacteria growth was seen in 10.8% of the cases. The OTU co-occurrence analysis displays the strong correlation between the presence of *F. nucleatum* and *P. intermedia*. Fusobacteria are present in apical abscesses because they constitute an important part of the apical biofilm [27, 32]. In particular *F. nucleatum* was frequently isolated and cultured from teeth with apical periodontitis and its virulence is greatly enhanced in presence of *P. intermedia* [33]*.*

Predicted metagenomes confirmed differences between the two types of samples and indicated that the RCs samples displayed a higher abundance of presumptive predicted metabolic pathways related to Lipopolysaccharide biosynthesis. This metabolic pathway is indicative of the presence of Gram negative bacteria and it is widely accepted as a subclinical pro inflammation marker [34]. The putative role of bacterial endotoxins in supporting epithelial proliferation typical of RCs has already been reported [35]. According to Meghji et al. [35], when epithelial cell proliferation assays were performed, the Lipopolysaccharides derived form three different bacteria displayed mitogenic effects, which were even enhanced if cyst fibroblast culture media were used.

## Conclusions

The present research, albeit preliminary, may contribute to the progress of the existing knowledge concerning the microbiota within the two main apical periodontal lesions. The use of sophisticated and sensitive techniques allowed unprecedented results and could help elucidating the possible etiopathologic role of a complex microbial environment in either promoting or refraining the epithelial proliferation.

## Abbreviations

AP: Apical periodontitis
HTS: High throughput amplicon target sequencing
NSTI: Nearest-sequenced-taxon index
OTUs: Operational taxonomic unit
PCoA: Principal coordinate analysis
PGs: Periapical granulomas
RCs: Radicular cysts
